# Comparison of Diagnostic Performance of Three-Dimensional Positron Emission Mammography versus Whole Body Positron Emission Tomography in Breast Cancer

**DOI:** 10.1155/2017/5438395

**Published:** 2017-07-03

**Authors:** Dong Dai, Xiuyu Song, Man Wang, Lin Li, Wenchao Ma, Wengui Xu, Yunchuan Ma, Juntian Liu, Jin Zhang, Peifang Liu, Xiaoyue Gu, Yusheng Su

**Affiliations:** ^1^Department of Molecular Imaging and Nuclear Medicine and Key Laboratory of Cancer Prevention and Therapy and Key Laboratory of Breast Cancer Prevention and Therapy, Tianjin Medical University Cancer Institute and Hospital, National Clinical Research Center for Cancer, Tianjin 300060, China; ^2^PET Center of Xuan Wu Hospital, Capital Medical University, Beijing, China; ^3^Division of Nuclear Technology and Applications, Institute of High Energy Physics, Chinese Academy of Sciences, Beijing 100049, China; ^4^Beijing Engineering Research Center of Radiographic Techniques and Equipment, Beijing 100049, China

## Abstract

**Objective:**

To compare the diagnostic performance of three-dimensional (3D) positron emission mammography (PEM) versus whole body positron emission tomography (WBPET) for breast cancer.

**Methods:**

A total of 410 women with normal breast or benign or highly suspicious malignant tumors were randomized at 1 : 1 ratio to undergo 3D-PEM followed by WBPET or WBPET followed by 3D-PEM. Lumpectomy or mastectomy was performed on eligible participants after the scanning.

**Results:**

The sensitivity and specificity of 3D-PEM were 92.8% and 54.5%, respectively. WBPET showed a sensitivity of 95.7% and specificity of 56.8%. After exclusion of the patients with lesions beyond the detecting range of the 3D-PEM instrument, 3D-PEM showed higher sensitivity than WBPET (97.0% versus 95.5%,* P *= 0.913), particularly for small lesions (<1 cm) (72.0% versus 60.0%,* P *= 0.685).

**Conclusions:**

The 3D-PEM appears more sensitive to small lesions than WBPET but may fail to detect lesions that are beyond the detecting range. This study was approved by the Ethics Committee (E2012052) at the Tianjin Medical University Cancer Institute and Hospital (Tianjin, China). The instrument positron emission mammography (PEMi) was approved by China State Food and Drug Administration under the registration number 20153331166.

## 1. Introduction

Breast cancer is the most common type of cancer in women worldwide, and there were approximately 1.7 million new cases in 2012 [1]. In China, the incidence of breast cancer increases continuously for the past two decades, and the estimated incidence and mortality in 2013 was 25.89 and 6.56 cases per 100,000 women, respectively [2, 3]. Early diagnosis is the key to improve the prognosis and outcomes of patients with breast cancer. The Swedish randomized trials demonstrated that mammography screening reduced the mortality of breast cancer significantly [4]. In addition to mammography, mammary ultrasonography, and breast magnetic resonance imaging (MRI), whole body position emission tomography (WBPET) has been used for diagnosis and staging of breast cancer [5–8]. WBPET detects suspicious mammary lesions based on the unique biochemical characteristics of breast cancer. Malignant mammary lesions usually have a higher rate of glucose metabolism than normal or benign tumors, leading to a significantly greater accumulation of radiotracer labeled glucose analogues, such as ^18^F-fluorodeoxyglucose (^18^F-FDG), in malignant lesions, which can be detected by WBPET scanning [5–8].

FDG-WBPET scan appears to be particularly superior to the nonbiochemical techniques such as mammography and mammary ultrasonography in patients without obvious cancer-associated anatomical changes. FDG-WBPET scan can detect malignancies in patients with dense or scarring breast tissues, whereas those nonbiochemical techniques usually fail on those patients [9]. In a meta-analysis to systematically compare the diagnostic accuracy of ultrasonography, computed tomography, breast MRI, mammography, and FDG-WBPET in patients with suspected recurrent and/or metastatic breast cancer, Pan et al. found that breast FDG-WBPET showed the highest pooled sensitivity [10]. However, the relatively low spatial resolution of FDG-WBPET (4 mm to 7 mm) limits its use on staging breast cancer and particularly limits its value to detect small lesions or lymph node metastases in breast cancer [11, 12].

To improve the spatial resolution, positron emission mammography (PEM) has been developed recently by multiple research institutes and medical instrument industry [9, 13–23]. Aliaga et al. tested PEM on animal models of breast cancer and found that the potential spatial resolution of PEM was 1.8 mm [24]. In a recent meta-analysis, Caldarella et al. showed that the pooled sensitivity and specificity for PEM were 85% and 79%, respectively in women with suspicious breast lesions [25]. Recently, Yamamoto et al. compared the imaging sensitivity of PEM versus WBPET in relation to tumor size and found that PEM showed significantly higher imaging sensitivity (78.6%) than WBPET (47.6%), particularly for small size tumors [26]. Large-scale trial to compare diagnostic performance of PEM versus WBPET in Chinese women is still lacking. This study aims to fill this knowledge gap. Here, in this double-center study, participants with normal breast or benign or malignant tumors received three-dimensional PEM (3D-PEM) and WBPET scanning sequentially. Diagnostic performance of 3D-PEM and WBPET was evaluated by comparing the imaging diagnosis with histopathological diagnosis. The detector of the 3D-PEM instrument used in this study has an average intrinsic spatial resolution of 1.67 mm [27].

## 2. Methods

### 2.1. Study Design and Settings

This prospective, noninterventional, double-center, randomized clinical study was conducted in Tianjin Medical University Cancer Hospital and Xuanwu Hospital of Capital Medical University from August 2012 to March 2014. The study protocol was approved by the Institutional Review Boards of Tianjin Medical University and Capital Medical University. The study was conducted in compliance with the Declaration of Helsinki, the Good Clinical Practices, and relevant ethical guidelines.

### 2.2. Participants

Informed consent was obtained from all study participants. Eligible participants were aged 18 to 70 years with or without a family history of breast diseases and had normal mammary gland, benign, or highly suspicious malignant mammary tumor. The mammary condition was evaluated by clinical tests, mammography, and mammary ultrasonography. The exclusion criteria were pregnancy and breast feeding, previous surgery, chemotherapy, or radiotherapy to treat malignancy, low tolerance to WBPET or PEM, being unable to keep prone position, being unable to undergo surgery although having an indication of surgery, or being unsuitable for the study based on the judgment of participating investigators. At the enrollment interview, general clinical data were collected. Mammography and mammary ultrasonography were performed or the results of these tests were obtained from the participants if they took the tests within 3 months prior to the enrollment interview. Eligible participants were scheduled for PEM and WBPET within 30 days of the interview. After PEM and WBPET, lumpectomy or mastectomy was performed on the patients that were eligible for surgery based on physician's judgment. Mammary biopsy was conducted prior to the surgery. Surgical tissue specimens collected after lumpectomy or mastectomy were examined by pathologists.

### 2.3. 3D-PEM Scanning

**Figure 1:**
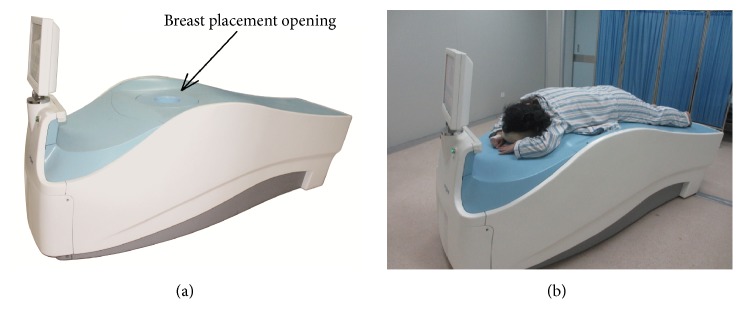
Images of the PEM system.

3D-PEM was performed using a PEMi-I scanning system (Gao Neng Medical Equipment Co., Ltd. Hangzhou, China). The PEMi-I system has a 64-ring detecting system, which allows for efficient acquisition of 3D images. The opening for breast placement has a diameter of 160 mm. The machine was designed for prone position, so that the breasts hang freely in the detector (Figure 1). Participants were required to fast for 4 to 6 hours and their fast blood glucose was determined. The radiotracer ^18^F-FDG (259–444 MBq) was injected intravenously to the participants that had a fast blood glucose level ≤ 140 mg/dL, and then the participants were required to rest for 50–60 minutes to allow the radiotracer to circulate. The participants that were allocated for the group of 3D-PEM followed by WBPET had 20-minute scan on the PEMi-I for each breast. Twelve-slice reconstructions were created, with slice thickness varying from 3 to 8 mm depending on breast thickness. Images were submitted to attenuation correction according to the image segmentation method. On the PEM images, breast tissue was separated from air based on the activity map of the breast. Linear attenuation coefficients (ACF) were obtained for each line of response based on the segmentation result. Reconstruction was repeated with the ACFs. WBPET was performed immediately after 3D-PEM (approximately 90 to 100 minutes after radiotracer injection).

### 2.4. Whole Body WBPET Scanning

Patients that were allocated to the group of WBPET followed by 3D-PEM underwent WBPET after radiotracer injection. WBPET was performed using the WBPET scanning system Discovery ST 4 UPG (GE, USA) or EXACT ECAT 47 (Siemens, Germany). The image acquisition (120 kV, 160–220 mA, helical pitch 0.75 : 1, and 5 mm slice thickness) was conducted using 2-minute emission acquisitions from the apex of the lung to the lower edge of the liver with participants at supine position. 3D-PEM was then performed immediately after WBPET (approximately 80–90 minutes after radiotracer injection).

### 2.5. Image Analysis

WBPET and PEM images were reviewed by 3 certified radiologists, who were blinded to participants' clinical information. A positive PEM and WBPET were defined as images showing continuous 2 layers of visible nodular or blocks of moderately to strongly abnormal radioactivity uptake. A negative PEM and WBPET were images showing no or very weak abnormal radioactivity uptake. Disagreements among the radiologists were discussed until reaching a consensus.

### 2.6. Surgical Histopathological Examination

Patients underwent lumpectomy or mastectomy within 1-2 weeks after WBPET and 3D-PEM examination. Surgical tissue specimens collected after lumpectomy or mastectomy were examined by pathologists. The histological grade and type were determined.

### 2.7. Evaluation

Concordant positive diagnosis was defined as the scanning images of both 3D-PEM and WBPET showing lesions with similar shape and size; concordant negative diagnosis represented absence of lesions on the scanning images of both 3D-PEM and WBPET. Positive WBPET was defined as images presenting more than 2 layers of visible nodular shaped or massive area of medium to severe abnormal increased uptake of radiotracer. Negative WBPET represented images showing uniformly distributed radioactivity or scattered, spotty, and mild increased uptake of radiotracer. The sensitivity and specificity of 3D-PEM and WBPET were compared. Calculations for concordance rate of positive diagnosis, concordance rate of negative diagnosis, overall diagnostic concordance, sensitivity, specificity, and accuracy are explained in Table 1.

### 2.8. Sample Size

At a significant level of 5% (2-sided), 239 participants with breast cancer were required to achieve 95% concordant positive diagnosis of 3D-PEM compared to WBPET with a power of 80%. Based on the assumption of 80% concordant negative diagnosis of 3D-PEM compared to WBPET at a significant level of 5% and a power of 80%, 153 participants with benign tumor or normal breasts were required. To achieve 90% overall diagnostic concordance of 3D-PEM compared to WBPET at a significant level of 5% and a power of 80%, 385 participants were required. Thus, the estimated total sample size was 392 (239 positive + 153 negative) participants, and a total of 400 participants (160 cases of negative malignancy + 240 cases of positive malignancy) were to be enrolled.

### 2.9. Randomization and Blinding

To minimize possible bias effects of the amount of ^18^F-FDG uptake on the diagnosis, participants were randomized at 1 : 1 ratio to undergo either 3D-PEM followed by WBPET (3D-PEM-WBPET) or WBPET followed by 3D-PEM (WBPET-3D-PEM). Randomization sequence was generated with the software SAS. Participants were not blinded for WBPET and 3D-PEM. The radiologists, who evaluated the scanning images of WBPET and 3D-PEM, were blinded for participants' clinical data.

### 2.10. Statistical Analysis

The statistical analysis was performed using the software SAS 9.13. Full analysis set (FAS) included data from participants with WBPET results. Per protocol set (PPS) included data from participants that were compliant with the study protocol, and participants with severe deviation from protocol, such as failure to undergo 3D-PEM, were excluded from PPS. Categorical variables are presented as percentage and continuous variables are presented as mean ± standard deviation (SD), median, minimum, and maximum. Diagnostic concordance, sensitivity, specificity, and accuracy were calculated. Student's* t*-test was used to compare patients' clinical characteristics. Chi-square test was used to compare sensitivity, specificity, and accuracy.

## 3. Results

### 3.1. Patient Flow and Baseline Data

**Figure 2:**
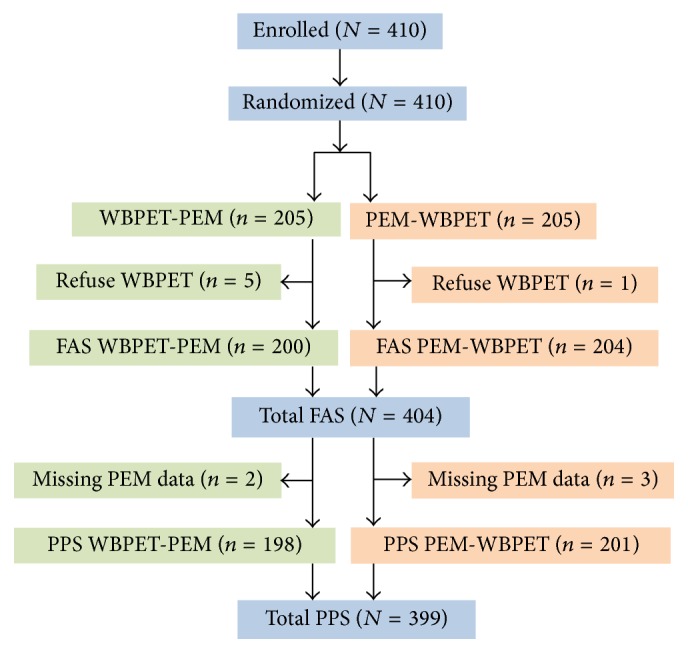
Patient flow chart.

A total of 410 participants, including 255 patients with highly suspicious malignancy based on mammography and mammary ultrasonography and 155 participants without malignancy were enrolled in the study and randomized to WBPET-3D-PEM or 3D-PEM-WBPET group. During the study, 6 subjects did not receive WBPET because of voluntary withdrawal from the study. Thus, FAS contained 404 participants. Among participants undergoing WBPET, 3D-PEM results were missing from 5 participants, resulting in PPS of 399 participants. Patient flow is displayed in Figure 2. Participants' baseline clinical data are described in Table 2. Clinical characteristics were comparable in participant receiving 3D-PEM-WBPET versus that receiving WBPET-3D-PEM (Table 2).

### 3.2. Evaluation

Diagnostic concordance of 3D-PEM and WBPET was analyzed on FAS and PPS data. For FAS data, concordance rate of positive diagnosis was 93.8% (95% CI: 90.6%–97.1%), concordance rate of negative diagnosis was 97.5% (95% CI: 94.8%–100.0%), and overall diagnostic concordance was 95.3% (95% CI: 93.1%–97.5%, Table 3). These results are similar to those from the analysis on PPS data (Table 3).

**Figure 3:**
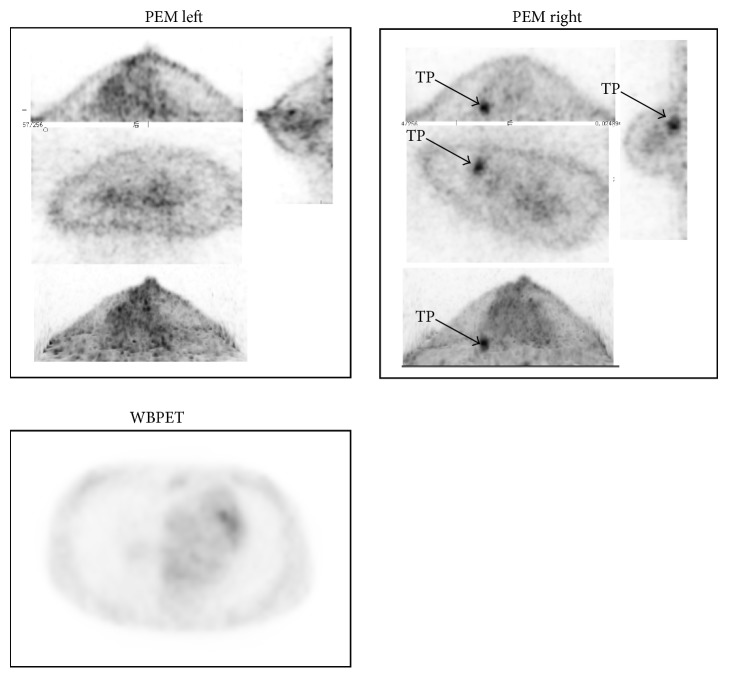
The images of the case showing false negative WBPET and true positive PEM. PEM images (left and right) and WBPET images of a 60-year-old woman with a true positive (TP) lesion (abnormal high ^18^F-FDG uptake, the maximum standard uptake value (SUVmax) is 4.12) in the right mammary gland (arrow pointing), whereas abnormal high ^18^F-FDG uptake was not shown in the PET (false negative, FN).

**Figure 4:**
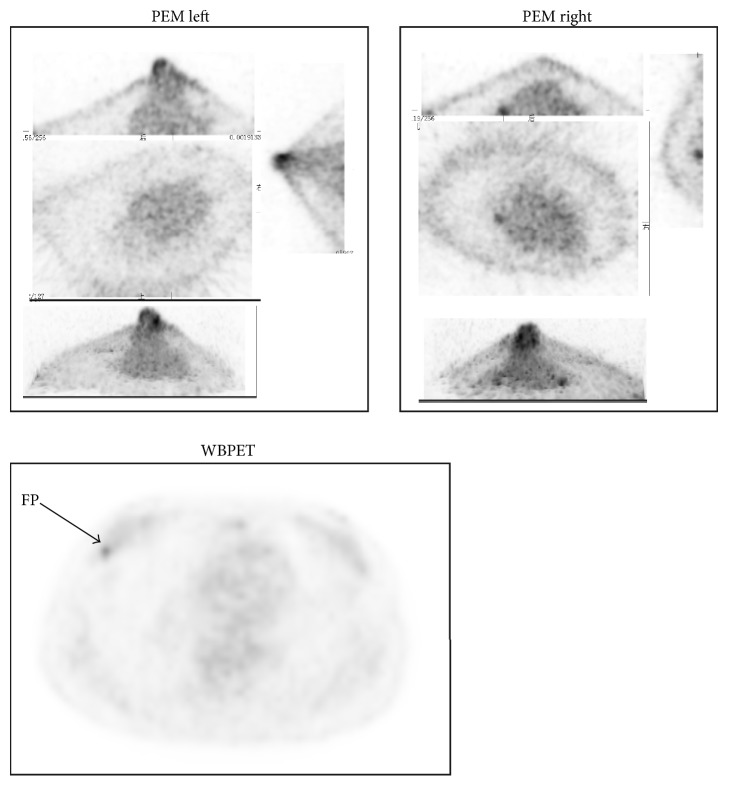
The images of the case showing false positive WBPET and true negative PEM. PEM images (left and right) and WBPET images of a 48-year-old woman without lesion (no abnormal high ^18^F-FDG uptake, true negative (TN)) in the left or the right mammary gland (arrow pointing), whereas abnormal high ^18^F-FDG uptake was shown in the PET (arrow pointing; the maximum standard uptake value (SUVmax) is 2.64; false positive (FP)).

A total of 19 participants showed inconsistent 3D-PEM and WBPET (Tables 3 and 4), among whom, 5 lost 3D-PEM data (Table 4). Histopathological examination of the remaining 14 cases revealed 3 cases of consistency between 3D-PEM and histopathological results and 11 cases of consistency between WBPET and histopathological results. Of the 11 cases of false diagnosis by 3D-PEM, 9 showed false negative 3D-PEM but true positive WBPET, and the lesions of the 9 cases were either near the chest wall (7 cases) or near the armpit (2 cases). These locations are out of the detecting range of the 3D-PEM detector (Table 4). The histopathological results of the 2 false positive 3D-PEM were one case of inflammatory lesion and one case of adenofibroma. Of the 3 cases wrongly diagnosed by WBPET, 2 were false positive and one was false negative. These 3 cases were accurately diagnosed by 3D-PEM. The 3D-PEM and WBPET scanning images of the case showing false negative WBPET and true positive 3D-PEM are presented in Figure 3. The images of the case with false positive WBPET and true negative PEM are presented in Figure 4.

The diagnostic accuracy of WBPET and 3D-PEM was evaluated using histopathology results as the gold diagnostic standard. Histopathology was available from 253 participants, including 209 malignant and 44 benign cases. The majority of the malignancy was infiltrating ductal carcinoma (159/209, 76.1%). There were only 18 cases (18/209, 8.5%) of ductal carcinoma in situ (DCIS), and the remaining cases (191/209, 91.5%) were invasive carcinomas. 3D-PEM and WBPET appeared to have similar specificity (54.5% versus 56.8%,* P* = 0.909) and accuracy (86.2% versus 88.9%,* P* = 0.808, Table 5). Although WBPET sensitivity (95.7%) was slightly higher than 3D-PEM sensitivity (92.8%), the values are not significantly different (*P* = 0.828, Table 5). To further estimate the performance of WBPET and 3D-PEM, we analyzed lesions < 1 cm and lesions ≥ 1 cm separately. Diameters of three dimensions were measured and the average diameter was calculated to represent lesion size. The mean lesion size (total 278 lesions) was 1.7 ± 8.4 cm. For the 44 small lesions (diameter < 1 cm), 3D-PEM showed higher sensitivity (69.2%) than WBPET (61.5%,* P* = 0.79, Table 5); for the 234 larger lesions (diameter ≥ 1 cm), WBPET sensitivity (92.1%) was slightly higher than PEM sensitivity (90.1%,* P* = 0.88, Table 5). However, sensitivity was not statistically significantly different between WBPET and 3D-PEM. No WBPET or PEM associated adverse event was reported during the study.

To accurately evaluate the diagnostic performance of 3D-PEM, we excluded the one patient with benign lesion out of PEM detecting range and the 9 patients, whose malignant lesions were beyond the range of PEM detector. We then compared the performance of WBPET and 3D-PEM on the 243 cases (253 − 10). The overall sensitivity of 3D-PEM (97.0%) was slightly higher than that (95.5%) of WBPET (*P* = 0.913, Table 6). In both small lesion (<1 cm) and large lesion (≥1 cm) subgroups, 3D-PEM sensitivity was higher than WBPET sensitivity (small lesions: 72.0% versus 60.0%,* P* = 0.685; large lesions: 93.8% versus 91.8%,* P* = 0.835, Table 6).

## 4. Discussion

In the current study, the diagnostic concordance of 3D-PEM and WBPET was higher than 95%. Since the first report on PEM by Thompson et al. in 1994 [14], several pilot clinical studies including small number of patients showed promising results of using PEM to diagnose breast cancer [21–23]. Levine et al. evaluated PEM on 18 biopsy-proven malignant lesions and found that PEM yielded a sensitivity, specificity, and overall diagnostic accuracy of 86%, 91%, and 89%, respectively [21]. Similarly, Rosen et al. tested PEM on 18 malignant and 2 benign mammary abnormalities and found a sensitivity of 86% [22], and Tafra et al. demonstrated that PEM led to a sensitivity of 87% in 44 newly diagnosed breast cancer patients [23]. In a recent meta-analysis to investigate the diagnostic accuracy of PEM to detect malignancy in women with suspicious breast cancer, Caldarella et al. found that the pooled sensitivity and specificity were 85% (95% CI: 83%–88%) and 79% (95% CI: 74%–83%), respectively [25]. However, only 8 studies were included in the meta-analysis and significant study heterogeneity was associated with the pooled sensitivity and specificity [25]. Compared with those previous reports, this current study showed a higher sensitivity of PEM, which was 92.8% in the 253 patients with histopathologically confirmed diagnosis and 97.0% in the 243 patients with lesions within the 3D-PEM detecting range. The higher sensitivity of PEM observed in this current study may be partially attributable to the high proportion of invasive breast cancers (>90%) in the patients. Caldarella et al. reported that the pooled sensitivity of PEM was higher (86%) for invasive cancers than for in situ cancers (81%) [25].

Because of the higher spatial resolution of PEM than WBPET, PEM is predicted to be more sensitive to detecting malignancies than WBPET, particularly for small size lesions [14]. Data from previous studies appear to support this prediction [26, 28]. In a recent report, Yamamoto et al. investigated the association between tumor size and the sensitivity of PEM and WBPET in 45 Japanese women with histopathologically confirmed mammary malignancy [26]. They found that PEM was significantly more sensitive than WBPET (66.7% versus 13.3%,* P* = 0.008) for lesions < 1 cm, whereas detection sensitivity for lesions ≥ 1 cm was comparable in the 2 imaging approaches [26]. They also showed that the sensitivity advantage of PEM over WBPET diminished as the lesion size increased [26]. Similarly, Schilling et al. reported that PEM had a significantly higher lesion detection sensitivity than WBPET (92.8% versus 67.9%,* P *< 0.001) [28]. Report by Kalinyak et al. also shows a significantly higher sensitivity of PEM to detect tumor in 69 patients with newly diagnosed breast cancer than WBPET (92% versus 56%) [29]. The current study also demonstrated that the PEM showed higher sensitivity than WBPET in small lesions, although the difference was not statistically significant because of the relatively low number of small lesions. Compared with the previously reported sensitivity of WBPET in breast cancer, which was between 64% and 96% [30], the detection sensitivity of WBPET in this current study (96.5%) is on the high end of the range. The relatively large average size of lesions in our patients (diameter > 1.5 cm) may contribute to the high sensitivity of WBPET. In addition, the WBPET systems used in this current study are dual time point imaging WBPET, which has been shown to have an improved sensitivity to detect invasive mammary malignancies [31].

This current study found 9 cases of false negative 3D-PEM, which were true positive from WBPET and histopathological analysis. Schilling et al. suggested that the false negative PEM in their study could be related to insufficient FDG uptake of the small size lesions [28]. Insufficient FDG uptake appeared to be not the reason for false negative PEM in this current study because the 9 cases were correctly diagnosed by WBPET. Careful review of the WBPET images revealed that the lesions of the 9 cases are beyond the detecting range of the 3D-PEM instrument. Of the 9 cases of false negative 3D-PEM, 7 lesions are next to the pectoral muscle and 2 lesions are near the armpit. In addition to very small size lesions with inadequate radiotracer uptake, the limitation of field-of-view associated with PEM instrument is also considered a major source of false negative results [25]. Deep small lesions located near to the pectoral muscle or in the axillary region are particularly difficult to be detected by PEM. The 9 false negative 3D-PEM cases may also contribute to the slightly lower overall sensitivity of 3D-PEM (92.8%) compared with WBPET (95.7%) in this current study. After exclusion of the 9 false negative 3D-PEM cases and the one case of benign lesion that was out of the detecting range of the 3D-PEM, the 3D-PEM showed a higher sensitivity than WBPET for all the lesions (97.0% versus 95.5%), small lesions (72.0% versus 60.0%), and large lesions (93.8% versus 91.8%). Histopathological analysis of the 2 false positive PEM cases revealed that they are one case of inflammatory lesion and one case of adenofibroma, suggesting that benign mammary abnormalities might also have a higher metabolic rate of glucose than normal breast tissue.

## 5. Conclusion

This current study found that 3D-PEM and WBPET showed satisfactory diagnostic concordance in Chinese patients and that the 3D-PEM appeared to be more sensitive than WBPET for lesions within the detecting range of the 3D-PEM instrument, particularly for small lesions with a diameter < 1 cm. The 3D-PEM instrument used in the current study may not detect lesions beyond the detecting range, particularly the regions near to the pectoral muscle and the axillary regions.

## Figures and Tables

**Table 1:** Calculation of diagnostic concordance and performance of WBPET and 3D-PEM.

	WBPET+	WBPET−
3D-PEM+	*A*	*B*
3D-PEM−	*C*	*D*

	Histopathology +	Histopathology −

3D-PEM or WBPET+	*a*	*b*
3D-PEM or WBPET−	*c*	*d*

Concordance rate of positive diagnosis of 3D-PEM compared with WBPET = *A*/(*A* + *C*) × 100%; concordance rate of negative diagnosis of 3D-PEM compared with WBPET = *D*/(*B* + *D*) × 100%; overall diagnostic concordance = (*A* + *D*)/(*A* + *B* + *C* + *D*) × 100%; sensitivity = *a*/(*a* + *c*) × 100%; specificity = *d*/(*b* + *d*) × 100%; accuracy = (*a* + *d*)/(*a* + *b* + *c* + *d*) × 100%.

**Table 2:** Baseline clinical characteristics.

	3D-PEM-WBPET (*n* = 204)	WBPET-3D-PEM (*n* = 200)	*P* value	Total (*N* = 404)
Age (years)				
Mean ± SD	50.1 ± 9.3	51.1 ± 9.1	0.2663	50.6 ± 9.2
Median (min, max)	50.0 (19.0, 71.0)	51.0 (20.0, 70.0)		50.0 (19.0, 71.0)
BMI (kg/m^2^)				
Mean ± SD	24.3 ± 3.4	24.5 ± 3.7	0.6061	24.4 ± 3.6
Median (min, max)	24.1 (17.3, 39.7)	24.2 (15.9, 43.0)		24.2 (15.9, 43.0)
SBP (mmHg)				
Mean ± SD	120.3 ± 12.1	122.5 ± 15.3	0.1075	121.4 ± 13.8
Median (min, max)	120.0 (87.0, 160.0)	120.0 (87.0, 180.0)		120.0 (87.0, 180.0)
DBP (mmHg)				
Mean ± SD	77.4 ± 7.9	78.1 ± 8.6	0.3913	77.7 ± 8.2
Median (min, max)	80.0 (51.0, 109.0)	80.0 (53.0, 100.0)		80.0 (51.0, 109.0)
Comorbidities, *n*				
Diabetes mellitus	9.7% (11/113)	11.7 (13/111)	0.6323	
Uterine fibroids	48.9% (23/47)	55.9% (19/34)	0.5366	

SBP: systolic blood pressure; DBP: diastolic blood pressure; SD: standard deviation; BMI: body mass index. Values in the 2 groups were compared by Student's *t*-test or chi-square or Fisher's exact test.

**Table 3:** Diagnostic concordance of 3D-PEM and WBPET.

	Full analysis set		Total
	WBPET +	WBPET −	
3D-PEM +	228	4	
3D-PEM −	15	157	
	243	161	404
Concordance rate of positive diagnosis: 228/243 = 93.8% (95% CI: 90.6%–97.1%)			
Concordance rate of negative diagnosis: 157/161 = 97.5% (95% CI: 94.8%–100.0%)			
Overall diagnostic concordance: 385/404 = 95.3% (95% CI: 93.1%–97.5%)			

	Perprotocol set		Total
	WBPET +	WBPET −	

3D-PEM +	228	3	
3D-PEM −	11	157	
	239	160	399
Concordance rate of positive diagnosis: 228/239 = 95.4% (95% CI: 92.5%–98.3%)			
Concordance rate of negative diagnosis: 157/160 = 98.1% (95% CI: 95.7%–100.0%)			
Overall diagnostic concordance: 385/399 = 96.5% (95% CI: 94.6%–98.4%)			

CI: confidential interval.

**Table 4:** Participants with inconsistent diagnosis from 3D-PEM and WBPET.

Subject ID	Age (years)	WBPET (left/right)	3D-PEM (left/right)	Histopathology (left/right)
3D-PEM loss				

004	46	+/−	NA/NA (loss)	+/NA
008	47	−/−	NA/NA (loss)	+/NA
010	58	−/+	NA/NA (loss)	NA/−
250	64	−/+	NA/NA (loss)	NA/+
251	47	+/−	NA/NA (loss)	+/NA

Consistent 3D-PEM and histopathology (false diagnosis of WBPET)				

003	35	−/+	−/−	NA/−
161	65	−/−	−/+	NA/+
128	45	−/+	−/−	−/−

Consistent WBPET and histopathology (false diagnosis of 3D-PEM)				

False negative 3D-PEM				
036	45	+/−	−/−	+/NA
063	38	−/+	−/−	NA/+
086	45	−/+	−/−	NA/+
089	46	−/+	−/−	NA/+
096	44	−/+	−/−	NA/+
244	48	+/−	−/−	+/NA
414	52	−/+	−/−	NA/+
177	55	+/−	−/−	+/NA
181	47	−/+	−/−	NA/+

False positive 3D-PEM				
018	44	−/−	+/−	−/NA
112	54	−/−	+/−	−/NA

+/+: left breast + and right breast +; +/−: left breast + and right breast −; −/+: left breast − and right breast +; −/−: left breast − and right breast −. NA: not applied (no histopathology examination). A positive test represents at least one side breast showing +. A negative test represents both sides showing −. Participants without 3D-PEM were considered to show inconsistent results compared with WBPET.

**Table 5:** Comparison of diagnostic performance of 3D-PEM and WBPET.

Total cases					
	WBPET *n* = 253		3D-PEM *n* = 253		*P* value
	+	−	+	−	

Histopathology +, *n* = 209	200	9	194	15	
Histopathology −, *n* = 44	19	25	20	24	
Sensitivity (%)	95.7		92.8		0.828
Specificity (%)	56.8		54.5		0.909
Accuracy (%)	88.9		86.2		0.808

	WBPET *n* = 44		3D-PEM *n* = 44		*P* value
	+	−	+	−	

Lesion < 1 cm					
Histopathology +, *n* = 26	16	10	18	8	
Histopathology −, *n* = 18	5	13	6	12	
Sensitivity (%)	61.5		69.2		0.79
Specificity (%)	72.2		66.7		0.878
Accuracy (%)	65.9		68.1		0.92

	WBPET *n* = 234		3D-PEM *n* = 234		*P* value
	+	−	+	−	

Lesion ≥ 1 cm					
Histopathology +, *n* = 203	187	16	183	20	
Histopathology −, *n* = 31	15	16	15	16	
Sensitivity (%)	92.1		90.1		0.88
Specificity (%)	51.6		51.6		1.0
Accuracy (%)	86.8		85.0		0.65

Values were compared by chi-square test.

**Table 6:** Comparison of diagnostic performance of 3D-PEM and WBPET after exclusion of lesions beyond the range of 3D-PEM detector.

Total cases					
	WBPET *n* = 243		3D-PEM *n* = 243		*P* value
	+	−	+	−	

Histopathology +, *n* = 200	191	9	194	6	
Histopathology −, *n* = 43	18	25	20	23	
Sensitivity (%)	95.5		97.0		0.913
Specificity (%)	58.1		53.5		0.517
Accuracy (%)	88.9		89.3		1.0

	WBPET *n* = 43		3D-PEM *n* = 43		*P* value
	+	−	+	−	

Lesion < 1 cm					
Histopathology +, *n* = 25	15	10	18	7	
Histopathology −, *n* = 18	5	13	6	12	
Sensitivity (%)	60.0		72.0		0.685
Specificity (%)	72.2		66.7		0.878
Accuracy (%)	65.1		69.8		0.613

	WBPET *n* = 225		3D-PEM *n* = 225		*P* value
	+	−	+	−	

Lesion ≥ 1 cm					
Histopathology +, *n* = 195	179	16	183	12	
Histopathology −, *n* = 30	14	16	15	15	
Sensitivity (%)	91.8		93.8		0.835
Specificity (%)	53.3		50.0		0.884
Accuracy (%)	86.7		88.0		0.912
